# Supplementary material for: Identification of photoperiod-induced specific miRNAs in the adrenal glands of Sunite sheep (Ovis aries)
Source: Front Vet Sci. 2022 Jul 22;9:888207. doi: 10.3389/fvets.2022.888207 (PMC9354845; doi:10.3389/fvets.2022.888207)
Supplement: Supplementary file 1 [file Data_Sheet_1.ZIP › Supplementary file/miRDeep2 file.zip!/miRDeep2 file/Know_Structure/SPLP42A2/oar-mir-323c.pdf]

miRBase precursor	: oar-mir-323c
Total read count	: 170
oar-miR-323c read count	: 167
remaining reads	: 3

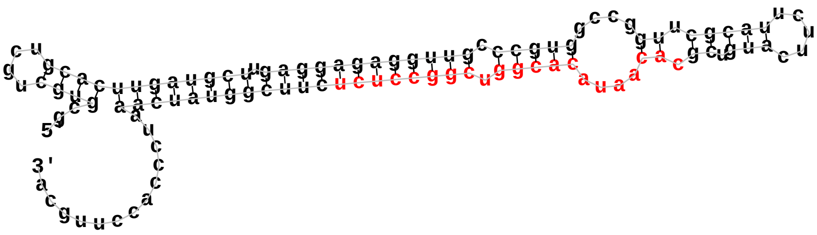

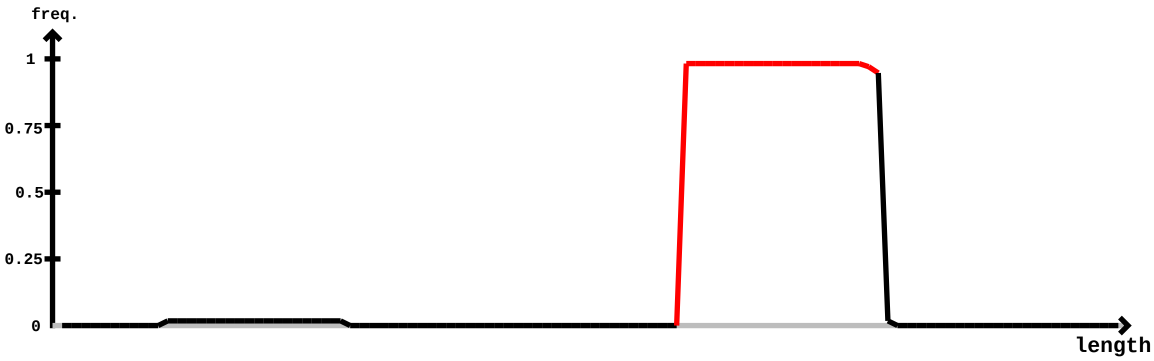

oar-miR-323c

```
5'- gcgugcugcugcacuugaugcuugaggagagguugcccguggccgguucgcauucuucaugucgcacaauacacggucggccucucuucgguaucaaaucccaccuugca   -3'    exp
    ..((((....))))((((((((.((((((((((((.(((((....((.(((((.....))).)).))....))))).))))))))))))))))))))............   reads      mm         sample
    .........gcacuugaugcuugaggag..................................................................................   3          0          seq
    ................................................................cacaauacacggucggccu...........................   2          0          seq
    ................................................................cacaauacacggucggccuc..........................   4          0          seq
    ................................................................cacaauacacggucggccucu.........................   158        0          seq
    ................................................................cacaauacacggucggccucuc........................   3          0          seq
```
